# Changing demographic trends among South African occupational therapists: 2002 to 2018

**DOI:** 10.1186/s12960-020-0464-3

**Published:** 2020-03-20

**Authors:** Lieketseng Ned, Ritika Tiwari, Helen Buchanan, Lana Van Niekerk, Kate Sherry, Usuf Chikte

**Affiliations:** 1 Centre for Rehabilitation Studies, Department of Global Health, Faculty of Medicine and Health Sciences, Stellenbosch University, Francie Van Zijl Dr., Tygerberg, Cape Town, 7505 South Africa; 2 Division of Health Systems and Public Health, Department of Global Health, Stellenbosch University, Cape Town, Western Cape South Africa; 3 Department of Health and Rehabilitation Sciences, University of Cape Town, Cape Town, Western Cape South Africa; 4 Occupational Therapy Division, Department of Health and Rehabilitation Sciences, Stellenbosch University, Cape Town, Western Cape South Africa; 5 Rural Rehab South Africa, Cape Town, South Africa

## Abstract

**Background:**

South Africa’s quadruple burden of disease, coupled with health system challenges and other factors, predicts a high burden of disability within the population. Human Resources for Health policy and planning need to take account of this challenge. Occupational therapists are part of the health rehabilitation team, and their supply and status in the workforce need to be better understood.

**Methods:**

The study was a retrospective record-based review of the Health Professions Council of South Africa database from 2002 to 2018. The data obtained from the Health Professions Council of South Africa was analysed for the following variables: geographical location, population groups, age, practice type and sex. Data was entered on a Microsoft Excel spreadsheet and analysed using the Statistical Package for the Social Sciences (SPSS version 22.0).

**Results:**

In 2018, there were 5180 occupational therapists registered with the Health Professions Council of South Africa with a ratio of 0.9 occupational therapists per 10 000 population. There has been an average annual increase of 7.1% over the time period of 2002–2018. The majority of occupational therapists are located in the more densely populated and urbanised provinces, namely Gauteng, Western Cape and KwaZulu-Natal. Most of the registered occupational therapists are under the age of 40 years (67.7%). The majority (66%) are classified as white followed by those classified as black and coloured. Females make up 95% of the registered occupational therapists. Nationally, 74.8% of occupational therapists are deployed in the private sector catering for 16% of the population while approximately 25.2% are employed in the public sector catering for 84% of the population.

**Conclusions:**

Under-resourcing and disparities in the profile and distribution of occupational therapy human resources remain an abiding concern which negatively impacts on rehabilitation service provision and equitable health and rehabilitation outcomes.

## Background

In South Africa, with a GINI coefficient of 0.63, inequity remains one of the defining features of the social, economic, political and health systems. The inequity at the level of health is stark when comparing the population dependent on the public sector (84%) and those with private medical insurance (16%), as well as differences between, and within, urban and rural public sector users. Health services play an important role in combating inequity, social injustice and the realisation of human rights. Human resources drive health system outcomes, and without an adequately staffed, competent, committed health workforce, the achievement of equitable health outcomes is compromised. The Sustainable Development Goals (SDGs) for 2030 cover a broad range of health challenges and emphasise the need to substantially increase health financing and the recruitment, development, training and retention of the health workforce.

The importance of the health workforce as a building block of the World Health Organization (WHO) health system framework is well-acknowledged globally [1]. The lack of trained manpower in disability and rehabilitation services inhibits the proper implementation and monitoring of all health system responsibilities. A critical mass of professionals involved with disability issues and/or rehabilitation is thus necessary to manage health systems and is often a crucial limiting factor in the delivery of quality rehabilitation services. Sustainable progress towards the realisation of Universal Health Coverage in South Africa as envisioned in the SDGs is imperilled with the lack of investment and development of Human Resources for Health (HRH) [2]. An ageing population coupled with a quadruple burden of disease contributes substantially to years lived with residual impairments in the general population [3–5]. Consequently, the demand, supply and scope for rehabilitation professionals are increasing. Such workers are not only an essential role in promoting health and improving quality of life, they also have an important role to play in managing the increasing burden of medicolegal costs faced by the state. A recent landmark judgement requires the state to provide the necessary health services to claimants in lieu of payouts for future medical expenses, provided such services are of equal quality to those available for purchase in the private sector [6]. The majority of such claims are for obstetric negligence leading to cerebral palsy. Occupational therapists are key service providers in the rehabilitation and long-term care of children and adults with cerebral palsy.

Despite these issues, human resources for the management of disability and rehabilitation services in South Africa (SA) lag behind the growing need for such services [5] which hampers service delivery [3]. Adequate provision of resources for rehabilitation services is an ineluctable part of health system strengthening [4].

The implementation of primary health care (PHC) re-engineering provided an opportunity for reconfiguring rehabilitation services as an integral part of health services that are offered as close as possible to where people live and work [7–9]. A service package for rehabilitation, however, has yet to be addressed and implemented comprehensively [9] as highlighted previously in an audit of national health care facilities which revealed that only 6–20% of PHC facilities offered rehabilitation services [10]. There is a paucity of data on the extent of the shortages of human resources and staffing norms for rehabilitation practitioners in the country [3]. The problem is compounded by inadequate rehabilitation units in district hospitals and the lack of rehabilitation services despite the policies on the re-engineering of PHC services. The current limited human resources for rehabilitation are subject to the same challenges experienced by other cadres of healthcare workers in the public sector [3] which include, but are not limited to, fiscal austerity, international migration, attrition, freezing of posts and movement to the private sector. The implementation of National Health Insurance (NHI) in South Africa has been accelerated as the strategy for achieving universal access to health care. The future health workforce would need to be geared towards enabling NHI to be implemented which has implications for every aspect of the health workforce as more health workers will be required, including at primary level and for rehabilitation services. A revision of the scope, roles and skills mix of practitioners, mid-level workers and community health workers needs to be articulated to refine the numbers needed. The needs will also be dependent on the package of services available to all South Africans and the quality of care offered. For example, if the primary care package is expanded and made more accessible, what increase can be forecasted for the number of health workers required in relation to disability services?

The recently published Framework and Strategy for Disability and Rehabilitation 2015–2030 in SA [5] also advocates for the need to improve human resources for disability and rehabilitation services, to truly transform the South African health system towards universal coverage and overcome existing inequities. The framework demonstrates the vacancy rate nationally at 22% with considerable variation across the provinces. The provinces of Limpopo (3%), Western Cape (5%), KwaZulu-Natal (12%) and North West (13%) had lower vacancy rates while the Eastern Cape (54%) and Mpumalanga (54%) had more than half their posts vacant. The vacancy rates for the provinces of Gauteng (16%), Northern Cape (28%) and the Free State (30%) were not inconsiderable. The findings suggest an inadequate distribution and high vacancy rate for all rehabilitation service providers at the different levels of care, but especially at the primary level [5].

Contributing to the vacancy rate is the widespread practice of freezing healthcare worker posts due to budget constraints and competing priorities in the Human Resources for Health Plan for South Africa [11]. This plan makes no mention of occupational therapists or any other rehabilitation therapists at a primary health care level, despite the intentions espoused by other policies, and this renders these professionals especially vulnerable to post-freezing.

It is also important to note that the vacancy rates reported are based on existing posts in the public health sector. To date, no standard methods exist for determining therapist staffing numbers in the public sector, and numbers of posts vary widely across different districts and institutions—often being historically and situationally determined, rather than by needs- or population-based formulae. Given the historical lack of investment in, and attention given to, rehabilitation in the health sector, it is reasonable to assume that the existing posts are considerably less than population needs.

Recently, the Academy of Science of South Africa (ASSAf) consensus study on health professional education compared graduate output for selected professionals during the period 2002–2010, with the increase in new appointments absorbed in the public sector [12]. The results have shown poor retention or absorption of health professionals (77.6% for occupational therapists) in the public sector. This concurs with the findings of other studies [13] that indicate that given the limited number of posts for rehabilitation therapists in the public sector, graduates often have a little option on completion of community service but to seek employment in the private sector [13].

Occupational therapy is a 4-year degree offered in eight higher education institutions in SA. Practitioners need to be registered with the Occupational Therapy, Medical Orthotics and Prosthetics and Arts Therapy Board of the Health Professions Council of South Africa (HPCSA) in accordance with the Health Professions Act No. 56 of 1974. Occupational therapists together with physiotherapists, speech-language therapists and audiologists constitute the professional groups making up the rehabilitation team in South Africa [5].

Occupational therapy services are delivered through both private and public sectors [14], playing an important role in addressing impairments associated with the burden of disease including maternal and child health (for example, birth trauma, cerebral palsy, developmental delays, mental illness, visual and hearing impairment), HIV and TB (neurological impairments, dementia, mental illnesses, TB of the spine, joint diseases, pain and fatigue, anti-retroviral side effects, ototoxic side-effects of TB medicines), trauma and violence (spinal cord injury, traumatic brain injury, amputation, orthopaedic complications, mental illnesses) and non-communicable diseases (stroke, diabetic retinopathy, neuropathies, amputation, mental illness, visual loss) [3]. Mental illness specifically is a major source of disability both in South Africa and worldwide in which occupational therapists play a crucial role. Occupational therapy services are delivered through both private and public sectors [14].

Subject to the rules of the HPCSA, occupational therapists can work in diverse sectors and settings such as health, education, labour (whether corporate, industrial, sheltered or informal), agriculture, traditional affairs, correctional services, transport, housing, mining, non-governmental organisations, organisations of people living with disabilities, retirement homes and frail care facilities, rehabilitation facilities and in people’s homes [15]. This is because occupational therapists are involved in all aspects of everyday living as shown in their scope of work. According to the draft regulations for the scope of the profession of occupational therapists in South Africa [15], occupational therapists enable participation and enhance performance in various occupation categories, namely education and learning, leisure, personal and community living, play, social participation and work. They also operate within four pre-occupational categories, namely biomechanical and neurological, psycho-social (including cognition, volition and affect), sensory and perceptual, and interpersonal—with clients across the lifespan [15].

Understanding the current supply and status of occupational therapists in the health workforce thus becomes imperative for policy-making that recognises the intersection between rehabilitation and other critical aspects of healthcare. Research into existing human resources in occupational therapy in South Africa is needed to assist in the planning of health worker training pipelines, service delivery and health budgets within the context of the goals expressed in universal health coverage (UHC) and the NHI framework. An assessment of the current occupational therapy health workforce will assist in the evaluation of shortages, enable the determination of gaps and provide a basis for forecasting optimal numbers as well as addressing what would constitute a core package for occupational therapy services. Therefore, the aim of this study was to describe the demographic trends of occupational therapists registered with the HPCSA from 2002 to 2018 as the first step towards understanding the supply and status of human resources for occupational therapy in South Africa.

## Methods

This was a retrospective record-based review of the HPCSA database from 2002 to 2018. A similar approach was adopted to that of a previous study [16] and relevant data recorded using a data collection sheet with the following variables: (i) category of health personnel (occupational therapy), (ii) category of practice, (iii) geographical location, (iv) population category and (v) sex. In this paper, we have used the term population group in line with the definitions in the Population Registration Act (Act No. 30 of 1950, 17) which previously classified South African citizens into four major population categories—‘white’, ‘coloured’, ‘Indian’ and ‘black’ [17]. Although the legislation was repealed in 1991, the population categories are still used in reporting in sectors such as the Department of Higher Education. Racial data remain important in monitoring the redress in the education and training of occupational therapists who were previously denied access to such training in terms of legislation.

The dataset was accessed, collected and analysed by the second author (RT), and accuracy was cross-checked by the first author (LN). Data were entered into a Microsoft Excel spreadsheet and analysed using the Statistical Package for the Social Sciences (SPSS version 22.0). Frequency distributions, cross-tabulations and graphical representations were used as descriptive statistical methods. Anonymity and confidentiality of all personnel were ensured as the data accessed from the HPCSA and presented in this paper is de-identified.

Data on occupational therapists currently employed in the public sector across various South African provinces were obtained from PERSAL (Personal and Salary System) 2018 data available in the 2018 South African Health Review (SAHR). The difference between the public sector figures from PERSAL and HPCSA registrations was calculated to obtain an approximation of the public-private spilt across the provinces. Ethical approval and a request for waiver of informed consent for this retrospective study were obtained from the Stellenbosch University Health Research Ethics Committee (HREC Reference No: X19/03/007) and the University of Cape Town Human Research Ethics Committee (HREC Reference No: 631/2019).

## Results

### Profile of occupational therapists

**Fig. 1:**
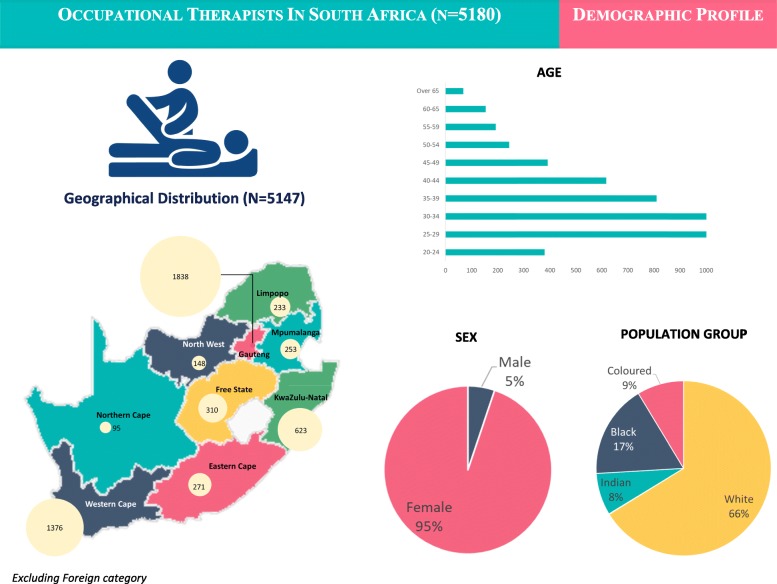
Profile of occupational therapists in South Africa (January 2018)

A total of 5180 occupational therapists were registered with the HPCSA in January 2018 with females comprising 95% (*n* = 4193) compared to 5% of males (*n* = 267). Fig. 1 provides a summary of the data according to geographical distribution, age, provincial distribution, age, sex and population categories.

### Growth in the number of occupational therapists

**Fig. 2:**
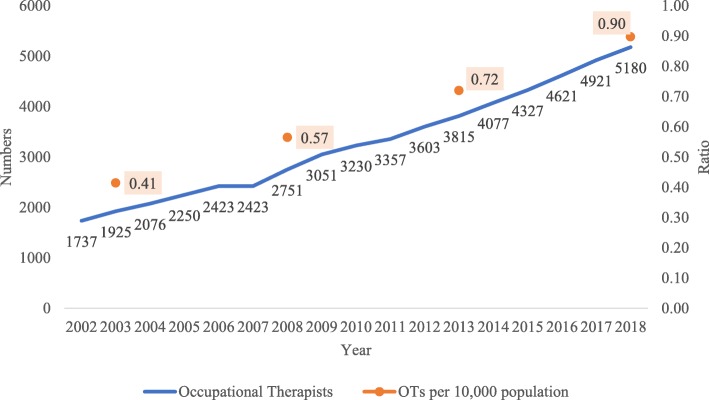
Registered occupational therapists and population ratios from 2002 to 2018

The number of occupational therapists has almost trebled from 2002 (*N* = 1737) to 2015 (*N* = 5180) with an average annual increase of 7.1%. As can be observed in Fig. 2, both the SA population and number of occupational therapists have increased over the 15-year period, compared with a 24.3% increase in the South African population. The ratio of occupational therapists per 10 000 population in SA also increased from 0.41 in 2003 to 0.90 in 2018.

#### Geographical distribution by province

The majority of occupational therapists were located in the more densely populated and urbanised provinces, namely Gauteng (35.5%), Western Cape (26.6%) and KwaZulu-Natal (12.0%). Free State (5.9%), Eastern Cape (5.2%), Mpumalanga (4.8%), Limpopo (4.5%) and Northern Cape (1.8%) hosted the lowest numbers of occupational therapists (see Table 1 and Fig. 1). Of these occupational therapists, a vast majority (89%) were registered in independent practice meaning they can practice autonomously in both the public and private sectors, while only 9% were registered in the public service category with restricted practice.

**Table 1:** National and provincial geographical distribution of occupational therapists

Province of employment	No. of occupational therapists	Percentage	Population %	Occupational therapists per 10 000 population
Gauteng	1 838	35.5	25.3	1.28
KwaZulu-Natal	623	12.0	19.6	0.56
Mpumalanga	253	4.9	7.9	0.56
Western Cape	1 376	26.6	11.5	2.11
Limpopo	233	4.5	10.2	0.40
Eastern Cape	271	5.2	11.5	0.42
North West	148	2.9	6.8	0.38
Free State	310	6.0	5.1	1.07
Northern Cape	95	1.8	2.1	0.80
Not applicable	33	0.6	–	–
Total	**5 180**	100	100	0.91

It is worth noting that the Eastern Cape and Western Cape have the same population, yet the Western Cape has five times the density of occupational therapists per 10 000 population than Eastern Cape. Although North West has half the population of the Western Cape, the density of occupational therapists is one fifth (0.38 per 10 000 population) of that of the Western Cape (2.11 per 10 000 population). The Northern Cape with the largest land mass and the smallest population has a density of 0.8 OTs per 10 000 people.

#### Age distribution of occupational therapists

Most registered occupational therapists were under the age of 40 years (67.7%). The number of registered occupational therapists dropped considerably after the age of 50 years.

#### Population category of occupational therapists

**Fig. 3:**
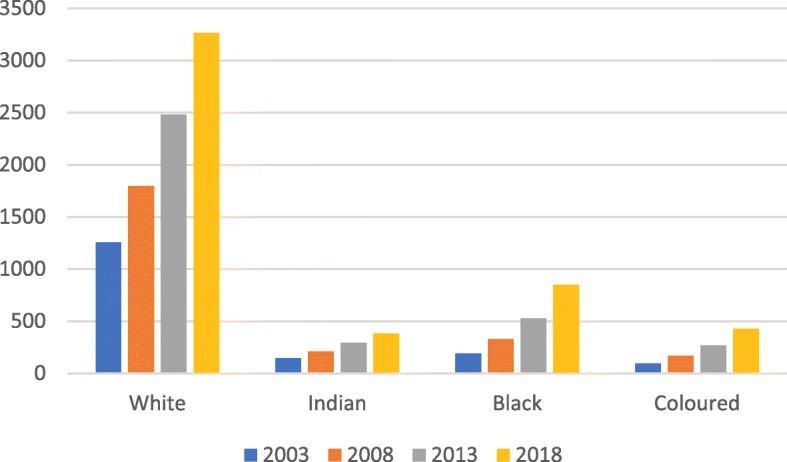
Distribution by population group over 5-year intervals

In 2018, the profile of registered occupational therapists in South Africa who identified as white was 65%, followed by black (17%), coloured (9%) and Indian (8%) (see Fig. 3).

The breakdown of registered occupational therapists for 5-year intervals starting in 2003 showed growth across all population groups.

#### Distribution of occupational therapists by sector (public versus private)

The number of occupational therapists across provinces working in the public sector as per SAHR 2018 [18] data compared with the numbers as per this HPCSA data gives some indication of the public-private split for occupational therapists at a provincial level. It is important to note however that ‘private’ in this instance may also include occupational therapists working for NGOs, those not currently in practice and those employed by the Departments of Education, Labour or Social Development.

**Fig. 4:**
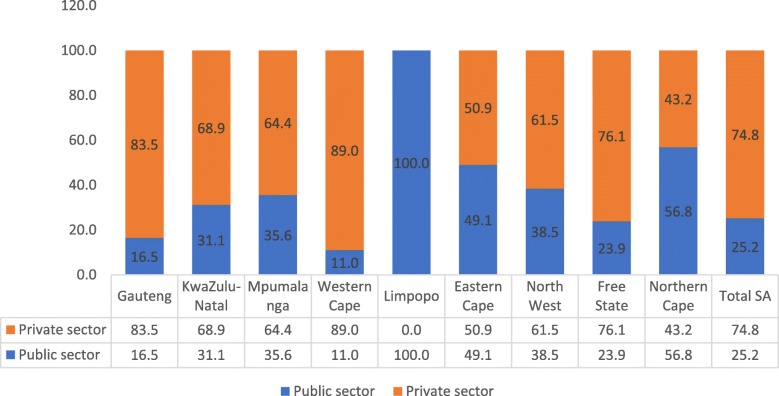
Percentage distribution of occupational therapists employed in the public and private sectors by province

The more urbanised and densely populated provinces of Gauteng (16.5%), Western Cape (11%) and KwaZulu-Natal (31.1%) have a lower proportion of occupational therapists employed in the public health service as compared to the other provinces in the country (see Fig. 4). Nationally, only 25% of occupational therapists were employed by the Department of Health.

#### Demographic trends by age, population group and sex

**Fig. 5:**
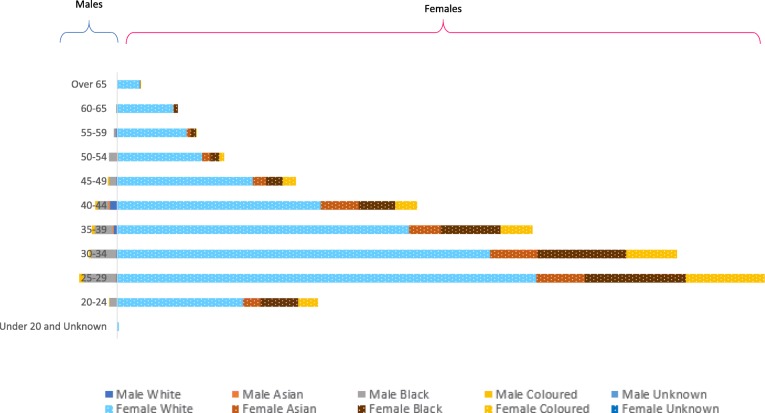
Breakdown of registered occupational therapists by age, sex and population group

Tracking demographic variables by age, population group and sex (see Fig. 5) showed that in 2018, the occupational therapy workforce was predominantly female and in the population group classified as white across all age groups. The number of occupational therapists decreased with age across all population groups; the decrease was noticeably higher in the section classified as white. Interestingly, the relatively few males in the profession were mostly younger and categorised as black.

**Fig. 6:**
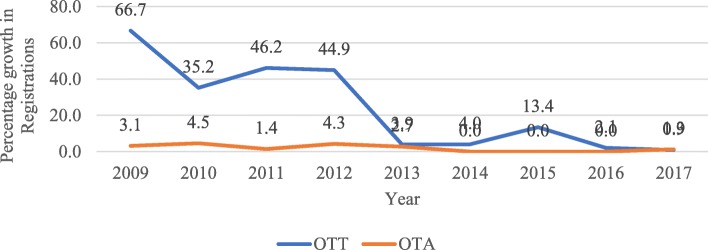
Percentage growth in OTT and OTA registrations (2009–2017)

The number of registered occupational therapy technicians (OTT) and occupational therapy assistants (OTA) has been on the decrease since 2009 (Fig. 6).

## Discussion

This study set out to describe the status of human resources for occupational therapy in South Africa. The profile of an occupational therapist employed in South Africa is likely to be female and classified as white in the 25–45 year age group, working in the private sector in one of the economically better-resourced provinces. The distribution trends across provinces in the 15-year period show that the three provinces (Gauteng, Western Cape and KwaZulu-Natal) have always enjoyed more resources, and little change has resulted from this position despite calls for a more equitable distribution of human resources. For instance, Gauteng had 39.4% distribution of occupational therapists in 2003, 37.4% in 2008, 36.7% in 2013 and 35.5% in 2018 while the province with the lowest distribution (Northern Cape) has not enjoyed much difference in this regard with percentages ranging from 0.6% in 2003, 0.6% in 2008 and 0.7% in 2013 to 0.6% again in 2018.

While growth in the number of occupational therapists was noted across all population groups, the breakdown showed the slow pace of transformation between population categories. Although most of the occupational therapy workforce is classified as white, the percentage growth within this category (as calculated over three 5-year periods) declined from 43.2% to 38% to 31.5%. During the last 5-year period (2013–2018), the growth of occupational therapists categorised as black was relatively high at 61.8%, followed by those categorised as coloured at 60.5%. The baseline for these two population categories was however extremely low. Occupational therapists classified as Indian and white grew at a rate of 31.1% and 31.5% respectively. While the segregated and discriminatory legislation and policies of selection practices for occupational therapy students have been abolished and the transformation efforts in the universities are noted, the factors that impede the recruitment and selection of a more diverse occupational therapy workforce are worth looking into. For instance, occupational therapy is less well-known than careers such as medicine and engineering, especially in rural areas. It also does not bring the same recognition or remuneration as other professional vocations but still demands high levels of academic ability. It is possible that candidates from previously disadvantaged backgrounds with the opportunity to enter professional training will prefer to choose careers offering higher status and earning potential. More research is needed however to understand the barriers to achieving a more racially representative occupational therapy workforce.

The largest increase by age occurred within the 25–29 and 30–34 age groups with a sharp decline from 35 years and even more so after 45 years (see Fig. 1). This decline could possibly be attributed to the poor retention and absorption rates with therapists emigrating or leaving the profession due to limited career pathing, job flexibility and earning potential within the public health system. Unfortunately, current evidence for this is anecdotal only, and more data is needed on both the earning levels and career choices of occupational therapists over time.

The ASSAf consensus study which compared the numbers of graduates for selected professions between 2002 and 2012 with the increase in the number of appointments in the public sector discovered a retention gap of 77.6% for occupational therapy [12]. Foreign-qualified occupational therapists could potentially contribute to the growth of the profession; however, they confront a complex administrative structure involving three stakeholders with systems that do not speak to each other. The first stakeholder is the HPCSA with its regulation stipulating completion of community service as a condition for registration. The second stakeholder is the Department of Health which does not make community service placements available to foreign qualified occupational therapists. The third stakeholder, the Foreign Workforce Management Programme, is involved in determining scarce skills required by the South African workforce. These complex structures make it challenging for foreign occupational therapists to become registered to practice.

The 2015 Global Burden of Disease Study (GBD) [19], the most consistent account of global, regional and national epidemiological evidence for all diseases and injuries in the current period, shows that of the total number of years lived with disability (YLD) in the world, 74% is associated with health conditions for which rehabilitation will be beneficial. Additionally, 15% of the total number of YLD globally results from health conditions such as epilepsy, multiple sclerosis and cancer, which are associated with severe impairments [18]. In South Africa, the HIV prevalence rate in 2018 was 13.1% while over the past 15 years, NCDs resulted in an estimated mortality rate of 37% and 16% of disability-adjusted life years [19].

A proxy indicator for the level of provision of rehabilitation services suggests there is a scarcity of rehabilitation specialists and that the assessed demand for rehabilitation is much higher than services can provide [20] especially in low- and lower-middle-income countries. Although there is no universally agreed or recommended minimum number for any of the rehabilitation specialists, the World Federation of Occupational Therapists (WFOT) suggests that the minimum number of occupational therapists should be 750 per one million population [21]. Data from 71 countries around the world show that the number of registered occupational therapists is far below this suggested minimum even in high-income countries [21]. While the number of occupational therapists in South Africa has increased by 12.5% since 2015 [22] along with a 5% population growth, the current ratio (2018) of 89.7 occupational therapists per one million population (pmp) in South Africa is eight times lower than that prescribed by the WFOT. The SA occupational therapy ratio pmp compares favourably with some lower- and upper-middle countries, such as Ghana (one pmp), Uganda (four pmp), Turkey (two pmp), Thailand (20 pmp), Kenya (20 pmp) and Tunisia (30 pmp), while countries such as Colombia (100 pmp) have considerably higher staffing ratios [23]. The South African Department of Health Workload Indicators of Staffing Need (WISN) tool arrived at a target ratio of 0.05 to 0.1 for PHC clinics and 0.1 to 0.1 for community health centres (CHCs) [24]. In terms of the WISN, there are currently 169 occupational therapists working in CHCs which means that an additional 238 are needed to meet the calculated target ratio of 407 overall need [24].

The WFOT ratios are currently not achievable and therefore not appropriate for SA within the current growth pattern fiscal space. This calls for a different approach for course correction of current HRH planning and policies for rehabilitation services. There is a need to develop clear staffing norms (which may serve as benchmarks) that could help to ensure equity in HRH distribution for occupational therapists. Thus, specific staffing norms will lead to specific service targets for specific health outcome targets in rehabilitation. It is suggested that norms are developed in ranges (minimum-maximum as per various scenarios) that may provide some room for flexibility in the system.

For HRH planning and estimation with regard to occupational therapy and other rehabilitation specialists, an estimated gap approach that considers needs may be better suited in the South African context [25]. The distributions of occupational therapy human resources noted earlier are not commensurate with the occurrence of disease and disability burden. For instance, while the Northern Cape province has the lowest percentage of occupational therapists (1.8%), it is also the province with the highest prevalence of disability (7%) [26]. Similarly, the Eastern Cape province, which has fewer resources, seems to have more challenges such as a higher infant mortality rate compared to the relatively better-resourced Western Cape [27]. Differences are also seen between urban and rural settings within these provinces [28] which could be explained by the fact that urban areas tend to have better human resources. This inequitable distribution of services makes sense in the light of the high proportion of occupational therapists employed in private or other non-governmental settings. Opportunities for such work are highest in urban centres, where more people are able to pay for private occupational therapy services. Difficulties in attracting and retaining healthcare workers in rural areas are widely acknowledged and apply equally to occupational therapists. Thus, even where public sector posts are funded in rural and low-socio-economic settings, it may be difficult to fill them.

Despite the global evidence showing that workforce drives the performance of the health system [29], responsive shifts and redistribution of human resources have not taken place in any significant manner. Compounding this issue could be factors such as the known poor retention, career pathing, freezing of vacancy posts, frustration related to working within the National Department of Health (NDOH) due to resource constraints as well as the political climate which has continued to lead occupational therapists to emigrate for more lucrative employment prospects. With such an unresponsive system, it is unsurprising to see from these findings that provinces with lower workforce seem to have poorer health outcomes. It is also worth mentioning that, together with the lower numbers of healthcare workers, poverty levels, unemployment and inequity are perhaps also more significant in driving poor health outcomes. Predominantly, rural provinces are poorer, have more problematic health services and also struggle to attract and retain staff in the public sector.

To date, it is only new graduates from the SA educational pipeline that contribute almost exclusively to the current growth of occupational therapists. Though community service was introduced to improve access to healthcare, we are however failing to retain these graduates post-community service as the ASSAf consensus study [7] has shown. There are very few plans aimed at recruitment and retention of occupational therapists beyond community service to the public sector. The lack of posts in the public sector is one of the primary reasons for poor retention of community service therapists [30]. Other push factors include restricted resources, poor supervision, cultural incompetence, feelings of unpreparedness for the skill set required [7, 9, 22, 31] negative exposure to rural and urban experiences during training, favouring urban over rural placements, proximity to home, family contact, relationships and dependents [32]. These factors require attention when developing a national human resource retention plan for public service.

Globally, occupational therapy has been a female-dominated profession. For example, in Canada, only 3% of all clinicians were estimated to be men [33]. There is a need for more men to join occupational therapy to serve the diverse clientele in hospitals, rehabilitation centres, career centres, nursing homes, schools, private practice and mental health facilities [34]. As a historically female profession, occupational therapy has also been politically, socially and economically disadvantaged. This is reflected in remuneration, status and recognition the profession enjoys, factors which act to maintain the marginalisation of rehabilitation broadly in the healthcare service and which could possibly explain the existing demographics of the profession.

Our findings show a rapid decline in mid-level workers (occupational therapy assistants and technicians). This is a concern because of mid-level workers’ well-recognised potential to broaden access to services and improve the quality of care. The main contributing factor for this decline is that there are currently no education programmes for occupational therapy mid-level workers in South Africa; this has been the case for more than a decade. As part of South Africa’s transition to democracy, the education landscape was re-structured to create the National Qualifications Framework (NQF) which aims to improve quality assurance, facilitate access, allow mobility and support progression in education. Towards this end, a single integrated framework comprising qualification levels and types was created. Unfortunately, there was no 2-year diploma in the first version of the NQF, thus forcing the discontinuation of programmes for occupational therapy technicians. A later version of the Higher Education Qualifications Sub-Framework now includes a 2-year qualification level, making the education of occupational therapy technicians possible. However, to date, none of the higher education institutions has started OTT training programmes despite efforts from the Occupational Therapy Association of South Africa (OTASA) to encourage them to do so.

It has been recognised that losing this cadre within the workforce is a problem. A survey at nine sites in South Africa found that disabled people living in sites with community rehabilitation workers had better access to healthcare services as well as health and education resources compared to those living in sites without this cadre of worker [35]. A systematic review on mid-level workers in South Australia showed the benefits of this workforce included improved clinical outcomes, increased patient satisfaction, higher-level services and more time for rehabilitation professionals to concentrate on patients with complex needs [36]. We therefore argue that the role and laddering of these mid-level workers could be a potentially cost-effective way of growing the number of occupational therapists in response to the rehabilitation needs as well as in supporting the current workforce.

Task shifting to community health workers (CHWs) will be the key to improving access to rehabilitation services to a greater portion of the population. In 2012, the Department of Health trained a new cadre of CHWs in the field of rehabilitation in order to improve access to health services to persons with disabilities. As per a study conducted in 2015, it was stated that the new cadre of rehabilitation care workers (RCWs) will strengthen rehabilitation services in intermediate care and in the community, and they will assist in promoting the participation of clients in the community [37]. In another study, rehabilitation health professionals (including occupational therapists) expressed their strong support for the utilisation of RCWs in intermediate care and in the community [38]. These RCWs help address the difficulties faced by persons with disabilities who often struggle to access health services [39]. Another promising development has been that of ward-based outreach teams (WBOTs) and strengthening the roles of CHWs which enables house-to-house visiting, offering excellent opportunities to identify people with disabilities in remote areas and to further link them with the necessary services [3]. However, this cadre may be overburdened as most professionals are trying to task shift their functions to them.

There is an evident imbalance in terms of the inter-provincial, inter-sectoral and urban versus rural distribution of occupational therapists. The data produced for this study are reliant on the accuracy of the records produced by the HPCSA. The HPCSA does not account for the sector in which health professionals work, and thus, inequity in access between the public and private sectors remains unreported [4]. Furthermore, the HPCSA does not report on emigration, death and retirement of health professionals or those who are registered but inactive [4].

## Conclusion

This research is timely as SA moves towards the implementation of the SDG with a particular focus on UHC under the umbrella of the NHI. HRH is a critical element to be considered in this context. Our findings show that over the 15 years studied, occupational therapists increased at a rate of 7.1%. Though this growth has exceeded that of the population, the density of occupational therapists to 10 000 population continues to be substantially lower than global recommendations. Therefore, it may take an unacceptably long time period to meet the demonstrated need which affirms the urgency to implement task-shifting to mid-level workers in order to meet the growing need for rehabilitation services. There is an urgent need to re-introduce OTT training.

Though the composition of the population groups of occupational therapists is changing, the historical inequalities remain entrenched. This slow pace of transformation does not match the needs of the people serviced. It is acknowledged that occupational therapy service users will be best served when the profile of the profession mirrors that of the general population because of language, culture and other contextual factors. Furthermore, the imbalance in the urban versus rural distribution of occupational therapists is problematic because occupational therapy’s main concern is re-establishing people in work, leisure, learning, play, personal and community living which require service users to have ready access to services close by. Furthermore, people in low-resourced contexts, who can least afford having to travel to access services, are disproportionately having to do so. For these reasons, the distribution of occupational therapists requires attention. Addressing the sex imbalance might lead to a politically stronger and better-recognised profession. Additionally, the registration for the foreign qualified workforce could be streamlined and simplified.

In a resource-deficit country like South Africa, there is a need for undertaking a granular HRH planning and forecasting exercise for rehabilitation services, focussing on more substantial elements such as disease burden, current fiscal space, and existing geo-spatial and social inequities to accessing rehabilitation services. Human resource strategies need to target the intractable health inequity generally with the aim to reduce the stark differences in the provision of health services and outcomes between the public and private sector. Limitations of this study include that the accuracy of HPCSA figures cannot be fully guaranteed. As such, there is much that this data cannot tell us—information which would influence demographic details such as where occupational therapists work and the sector of work. The existing data only described the current situation. Some of the key HRH aspects of the workforce are also unreported such as possible changes in the burden of disease, service delivery models, facility planning, facility workloads, skills mix, remuneration systems and health worker productivity, all of which are important in projecting future HRH needs and costs. The findings raise some concerning questions about the profession, specifically implications for access to rehabilitation services.

## Data Availability

The datasets generated and/or analysed during the current study are not publicly available.
